# Effect of Adding Silver-Doped Carbon Nanotube Fillers to Heat-Cured Acrylic Denture Base on Impact Strength, Microhardness, and Antimicrobial Activity: A Preliminary Study

**DOI:** 10.3390/polym15132976

**Published:** 2023-07-07

**Authors:** Abdulaziz Alhotan, Rasha M. Abdelraouf, Sabry A. El-Korashy, Nawaf Labban, Hanan Alotaibi, Jukka P. Matinlinna, Tamer M. Hamdy

**Affiliations:** 1 Department of Dental Health, College of Applied Medical Sciences, King Saud University, P.O. Box 10219, Riyadh 12372, Saudi Arabia; aalhotan@ksu.edu.sa; 2 Biomaterials Department, Faculty of Dentistry, Cairo University, Cairo 11553, Egypt; rasha.abdelraouf@dentistry.cu.edu.eg; 3 Department of Chemistry, Faculty of Science, Suez Canal University, Ismailia 41511, Egypt; selko58@yahoo.com; 4 Department of Prosthetic Dental Sciences, College of Dentistry, King Saud University, P.O. Box 60169, Riyadh 11545, Saudi Arabia; nalabban@ksu.edu.sa (N.L.); haalotaibi@ksu.edu.sa (H.A.); 5 Biomaterials Science, Division of Dentistry, Faculty of Biology, Medicine and Health, The University of Manchester, Manchester M13 9PL, UK; jukka.matinlinna@manchester.ac.uk; 6 Restorative and Dental Materials Department, Oral and Dental Research Institute, National Research Centre (NRC), El Bohouth St., Dokki, Giza 12622, Egypt

**Keywords:** poly (methyl methacrylate), PMMA, heat-cured acrylic resin, silver-doped, carbon nanotubes, CNT, impact strength, surface microhardness, antimicrobial activity

## Abstract

Poly (methyl methacrylate) (PMMA), is an acrylic polymer substance that is mostly used for denture base applications. The purpose of this laboratory study was to investigate the effect of adding 0.05 wt.% Ag-doped carbon nanotubes (CNT) to PMMA-based (PMMA and MMA) denture base material on the impact strength, microhardness, and antimicrobial activity. A total of 60 heat-cured acrylic resin specimens were prepared. The specimens were randomly divided into two main groups (*n* = 30/group), according to the powder used: (a) control group, using heat-cured PMMA; (b) treatment group, using a powder prepared by blending 0.05 wt.% silver-doped CNT nanoparticles with heat-cured PMMA. The impact strength, microhardness and anticandidal activity for each group were assessed via the Charpy, Vickers and agar diffusion tests, respectively (*n* = 10/test for each subgroup). Data were analyzed using independent-sample *t*-tests (*p* ≤ 0.05). The results of the impact strength test revealed that the treated heat-cured PMMA-MMA with Ag-doped CNT (2.2 kJ/mm^2^) was significantly higher than that of the control heat-cured PMMA (1.6 kJ/mm^2^). Similarly, the Vickers microhardness of the treatment group (52.7 VHN) was significantly higher than that of the control group (19.4 VHN). Regarding the agar diffusion test, after 24 h of incubation, the treated heat-cured PMMA with the Ag-doped CNT exhibited significantly higher anticandidal activity than that of the control group. Therefore, Ag-doped carbon nanotubes could be considered as promising fillers for the dental heat-cured acrylic resin to improve the resistance of the resultant denture against sudden fractures, scratching, and candida invasion.

## 1. Introduction

Synthetic acrylic polymers play a crucial role in the field of dentistry [1,2,3,4], offering excellent mechanical and biological properties that make them ideal for various dental applications [5]. Among these polymers, poly (methyl methacrylate) PMMA, is still the material of choice in dental clinics and laboratories [6]. In particular, conventional PMMA is commonly used for constructing removable denture bases. It possesses the advantage of easy manipulation, solidifying, and setting upon the polymerization reaction. Acrylic resins, available in heat- or chemical-cured forms, consist of a powder–liquid system. The powder comprises PMMA polymer beads, along with pigments, gums, fibers, and some other additives to control physical properties as well as enhance esthetic and optical properties. The liquid component contains methyl methacrylate, MMA, cross-linkers, activators, and inhibitors [6,7]. PMMA–MMA polymers are utilized in denture base fabrication, bone cements in orthopedics, and employed for denture repair [8,9]. The continuous popularity of PMMA in dental applications stems from its unique and beneficial properties, such as cost-effectiveness, ease in manipulation, acceptable mechanical properties, and the ability to be tailored to meet specific requirements [10,11,12]. Moreover, there are no alternatives for PMMA in the market. However, it is important to realize some drawbacks associated with PMMA, such as its insufficient surface microhardness, modulus of elasticity, flexural strength, and low impact strength [13,14].

Clinically, the most common form of failure in acrylic resin denture bases is a fracture caused by a sudden impact. Fractures can occur to the denture inside the patient’s mouth over long-term use because of the exposure to high levels of flexural stresses caused by biting. In addition, fractures can happen outside the patient’s mouth due to accidentally dropping the denture, highlighting the need to improve the impact strength [15,16]. There is a significant demand for enhancing the dentures mechanical properties. Increasing the surface microhardness of denture base materials facilitates easier finishing and improves resistance to scratching and abrasion during denture cleaning and maintenance. Reduced surface microhardness in acrylic denture bases increases the likelihood of abrasion and crack formation, which promote plaque and bacterial accumulation and weakens the denture base [17].

In recent years, nanotechnology has been contributing to dentistry, and numerous studies have been conducted to explore the potential benefits and implications [18]. Polymeric nanocomposites consist of nanoparticles incorporated as fillers within a polymeric matrix [10,19,20]. Some studies have demonstrated that incorporating nanoparticle fillers into a PMMA matrix can provide sophisticated and improved physical, mechanical, and antimicrobial properties, resulting in novel polymeric nanocomposites [21]. 

The need to improve the properties of acrylic resin denture base materials for biomedical applications has become a significant concern. In response to the increasing demand for enhanced denture base materials, several attempts have been made to improve the mechanical properties of PMMA. Some studies have reported the reinforcement of PMMA by using different fibers, ceramic particles, metallic nanoparticles, or even nanotubes, which have demonstrated some beneficial features [18,22,23,24,25,26,27]. Nevertheless, incorporating strong and hard nanoparticles into the polymeric matrix significantly enhances the mechanical and surface properties of the polymeric nanocomposite [24,25]. Some other investigations have focused on the addition of epoxy resins or polyamide materials [28,29].

Several studies have demonstrated that incorporating a significant amount of silver nanoparticles into polymeric PMMA composites exhibits antifungal activity without compromising the mechanical properties [30,31,32]. Carbon nanotubes, CNTs, are considered one of the most promising nanomaterials due to their high potential for biological applications, thanks to their improved mechanical and physico-chemical features. CNTs have a hollow cylindrical shape composed of hexagonal carbon rings [33]. Integrating nanoparticles with nanotubes aims to create nanoscale composites that are strong, hard, adaptable, lightweight, and cost-effective [34]. CNTs are already used as reinforcement fillers for polymers [35]. The intrinsic reinforcement behavior of nanotubes in polymers is achieved by optimizing the interaction at the interphase between the nanotube surface and the polymer, providing satisfactory adhesion, and facilitating the transfer of stresses from the flexible but weaker polymer to the more strong, rigid, and hard nanotubes, thereby reducing dimensional changes [36,37]. 

Compared with other elements in nature, carbon exhibits unique diversity in its ability to exist in various allomorphs and chemical structures, from carbon black to diamonds and polymers, with almost an infinite number of applications. CNTs can be employed as fillers in low concentrations for reinforcement and their ability to transfer loads at the interphase [38]. With features such as a high aspect ratio, ultra-light weight, hardness, high tensile strength, superior electrical conductivity, and chemical and thermal stability, CNTs have garnered significant interest in the fabrication of new materials [39].

Silver and its salts have long been utilized in medical applications due to their antibacterial properties. They are characterized by long-lasting bactericidal activity, low toxicity, and good biocompatibility. Ag nanoparticles are effective antibacterial fillers and also act as strengthening agents [40]. Doping CNTs with Ag nanoparticles can potentially create unique fillers with predictable superior reinforcement and antibacterial activity [39]. The objective of the current laboratory study was to investigate the effects of addition of Ag-doped CNTs to heat-cured acrylic denture bases and compare their impact strength, microhardness, and antimicrobial activity against a common oral yeast, *Candida albicans*. The null hypothesis was that an addition of 0.5 wt.% Ag-doped CNTs to heat-cured PMMA would not affect the impact strength, surface microhardness, and anti-candida activity, compared with the non-treatment group (control).

## 2. Materials and Methods

The present experimental study was approved by the Medical Research Ethical Committee (MREC) of National Research Centre (NRC), Cairo, Egypt (Ref. number: 243012023). The sample size was calculated using the G*Power (version 3.1.9.7) sample size calculator, based on means and standard deviations [34]. The estimated sample size in each group was 30, i.e., 10 for each test (i.e., impact strength, microhardness and agar diffusion tests).

### 2.1. Specimen Groups

A total of 60 specimens (*n* = 60) were prepared for this study using heat-cured poly (methyl methacrylate) (Acrostone Dental Manufacture, Cairo, Egypt). The specimens were randomly divided into two main groups (*n* = 30 per group) based on the type of powder used in the mixing process:

(a) The control group was prepared by mixing heat-cured PMMA powder with the monomer liquid.

(b) The treatment group was prepared by blending 0.05 wt.% Ag-doped carbon nanotube (CNT) nanoparticles into the heat-cured PMMA powder, followed by mixing with the monomer liquid.

### 2.2. Preparation of Ag-Doped CNT

Silver nanoparticles were prepared using the chemical reduction method by dissolving 50 mL of 0.001 M AgNO_3_ (Sigma-Aldrich, Munich, Germany) in bidistilled water, which was then heated to its boiling point using a hot plate with a magnetic stirrer. To this solution, 5 mL of 1% trisodium citrate was added dropwise. Heating was continued until the solution turned yellowish-brown (visually verified). Then, it was removed from the heating element and stirred until it reached room temperature [41]. The loading of Ag nanoparticles into CNT was performed by slowly adding 0.15 g of multiwalled CNT, with the average length of 18–25 nm (Sigma-Aldrich, Munich, Germany), into the solution with vigorous stirring for 1 h, which was then filtered and rinsed with distilled water several times and dried at 80 °C for 24 h.

To prepare the powder of the treatment group specimens, the resultant powder (silver-doped CNT) was added to the heat-cured PMMA powder (0.05 wt.%: 99.95 wt.% respectively) and mixed manually in a container for 10 min to guarantee an even distribution.

### 2.3. Specimen Preparation

**Figure 1:**
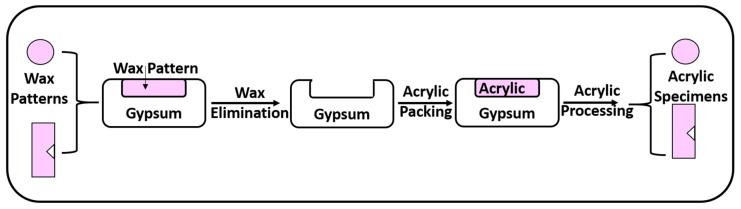
Schematic diagram of specimen preparation.

Figure 1 represents a schematic diagram showing the steps of specimen preparation.

(a) Construction of Wax Patterns

Teflon molds with a central hole (10 mm × 2 mm) were used to prepare disc wax patterns for the microhardness and anti-candidal tests. Meanwhile, for the impact strength test, a metallic stainless-steel mold (50 mm × 6 mm × 4 mm) with a 1.2 mm V-shaped projection was used to prepare rectangular specimens with a V-shaped notch (ISO 180:2019).

Pink wax (Cavex Holland, Haarlem, The Netherlands) was softened and adapted to fill the Teflon or metallic molds. A glass slab was placed on the mold’s top surface until the wax hardened. Next, the disc wax patterns were taken out from the mold.

(b) Flasking and Wax Elimination

Denture flasks were utilized to transform wax patterns into acrylic resin specimens. Gypsum (Gypsano, Cairo, Egypt) was mixed with water in accordance with the manufacturer’s instructions, and the resulting mix was poured to fill the lower half of the flask. The wax patterns were embedded into the soft gypsum. The gypsum was allowed to set.

After gypsum setting, separating medium (Acrostone, Cairo, Egypt) was applied with a brush to the top surfaces of the specimens and gypsum. After placing the upper half of the flask, gypsum was mixed as before and poured over them. Once filled, a cover was placed on the top of the flask and left in place to harden completely. The flasks were submerged in hot water at a temperature of 100 °C for 5 min to remove the wax. The flasks were opened, and the wax that had melted inside was removed, leaving empty molds.

(c) Preparation of Acrylic Resin Specimens

Using a stainless-steel spatula, the PMMA powder and liquid were mixed in a glass container in a 3:1 volume ratio. When the mixture reached the dough stage, it was packed into the mold spaces that had been previously prepared and then painted with a separating medium in the flask. The flask was subjected to pressure until metal-to-metal contact was reached. In an automated polymerization device (Kavo EWL 5501; Kavo Electrotechnisches Werk, Leutkirch, Germany), all discs were polymerized in water at 70 °C for 7 h and stored there at 100 °C for 3 h. The flask was allowed to slowly cool after curing. After finishing the de-flasking technique and removing any excess material with an acrylic bur, the specimens were ready. The discs were polished with pumice-filled polishing brushes after being smoothed with sandpaper. Distilled water was used to rinse the discs. Before testing, all the discs were cleansed ultrasonically in distilled water for 20 min and then kept in distilled water at 37 °C [42,43].

### 2.4. SEM and EDX Analysis of the Ag-Doped CNT and Specimens

SEM analysis (SEM; JSM-5200, JEOL, Tokyo, Japan) was performed for Ag-doped CNT and both untreated (control) and treated specimens with a working distance of 10 mm, magnification of 2000× and 5000×, resolution of 3 nm, and an accelerating voltage of 30 kV. An additional SEM micrograph was taken for the powder with the magnification 20,000×. 

Chemical analysis for the powder and the specimens was carried out, employing an environmental scanning electron microscope (SEM; JSM-5200, JEOL, Tokyo, Japan) and energy-dispersive X-ray spectroscopy (EDX; Oxford Inca Energy 350, Oxford Instruments, Abingdon, UK), with a working distance of 10 mm, resolution of 3 nm, and an accelerating voltage of 30 kV. The automatic identification of elements and element quantification in both wt.% and atomic % were performed following the collection of EDX spectra. SEM images and associated EDX spectra were carefully recorded.

### 2.5. Impact Strength Test

The impact strength was evaluated using the Charpy tester (Ceast-Resil impactor, Type 6967000, Turin, Italy). Each V-shape notched specimen was clamped horizontally from both ends, and a swinging pendulum was applied to hit in the center of the specimen to induce fracture. The scale readings on the fractured specimen were used to determine the energy absorption and impact energy (in J) of the specimen. The following equation was used to calculate the Charpy impact strength [44]:

Impact strength (kJ/m^2^) = E/TW

where E = the absorbed energy (kJ), W = the width of the specimen (m), and T = the thickness of the specimen at the notch base (m).

### 2.6. Microhardness Test

The Digital Vickers Hardness Tester (NEXUS 400TM, INNOVATEST, model 4503, Maastricht, The Netherlands) was used to measure the surface microhardness. The indentations were produced within 15 s of the loading 100 g at 20× magnification [17]. Vickers hardness indentations were made randomly at three different points on the specimen in each sample, and the mean Vickers hardness number (VHN) was calculated automatically using the following equation [17]:

VHN = 1.8544 P/d^2^

where P = the applied force (kg) and d = the mean of the two diagonals gained from the indentation (mm).

### 2.7. Anti-Candida Effect by Agar-Diffusion Test

Agar diffusion tests were performed by inserting the prepared discs into agar plates (Sabouraud Dextrose Agar) containing *Candida albicans*, then incubating at 37 °C, and measuring the inhibition zone in mm. The inhibition zone was calculated immediately after and after 24 h of incubation.

### 2.8. Statistical Analysis

The statistical analyses were conducted with statistics software (IBM-SPSS version 27.0, New York, NY, USA). The Kolmogorov–Smirnov and Shapiro–Wilk tests were all non-significant (*p* > 0.05 in all cases). This demonstrated that the data were relatively normally distributed. The impact strength, microhardness and agar diffusion test results were analysed and compared using independent samples *t*-tests. *p* ≤ 0.05 was adopted as the criterion for statistical significance.

## 3. Results

### 3.1. SEM and EDX of the Produced Ag-Doped CNT and Specimens

**Figure 2:**
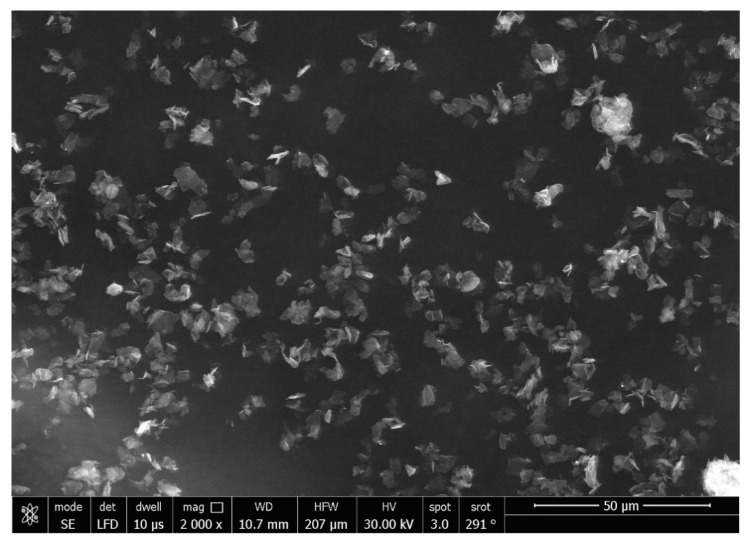
SEM micrograph of the Ag-doped CNT (magnification 2000×).

**Figure 3:**
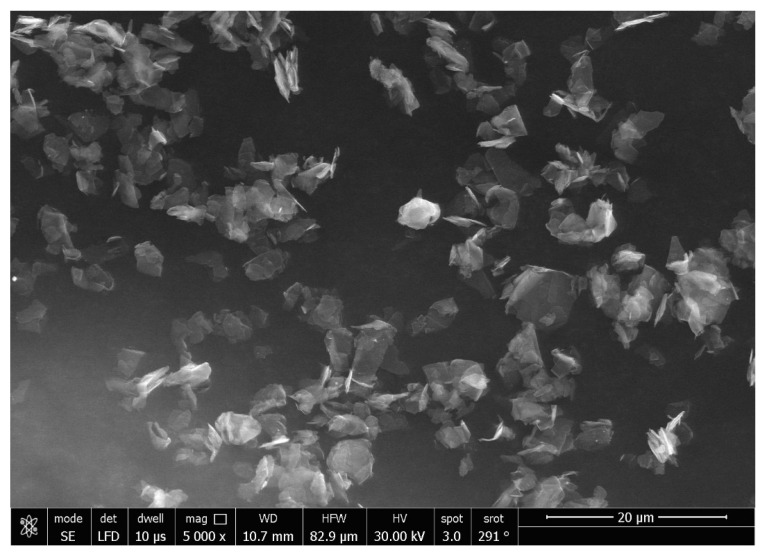
SEM micrograph of the Ag-doped CNT (magnification 5000×).

**Figure 4:**
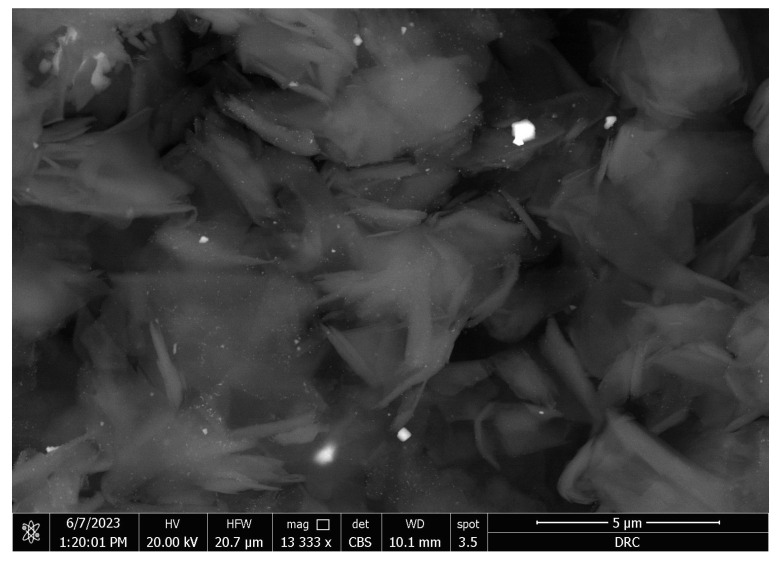
SEM micrograph of the Ag-doped CNT (magnification 20,000×).

**Figure 5:**
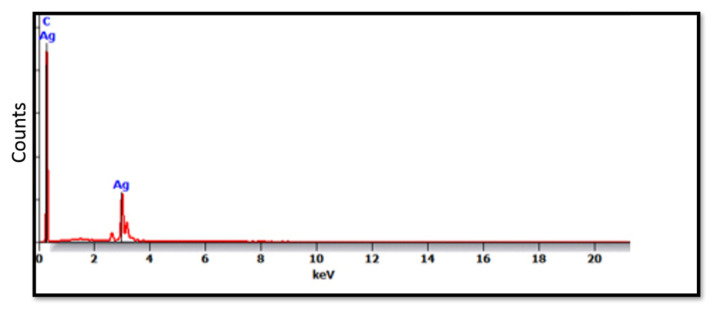
EDX spectrum of the Ag-doped CNT.

The SEM micrographs of the Ag-doped CNT are represented in Figure 2, Figure 3 and Figure 4 at the magnifications 2000×, 5000×, and 20,000× respectively. The SEM micrographs revealed an agglomerated structure with a flake-like appearance with an average size of 4.5 µm ± 0.5 µm × 2.5 µm ± 0.5 µm. The elemental analysis revealed that the composition of the powder was mainly Ag and C (wt.%: 57.9% and 40%, respectively, or in at.%: 16% and 84%, respectively) (see Figure 5).

**Figure 6:**
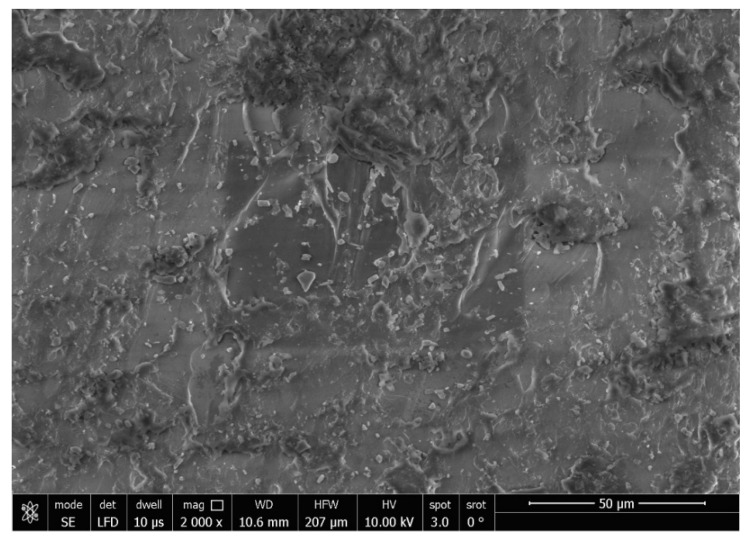
SEM micrograph of control specimen (magnification 2000×).

**Figure 7:**
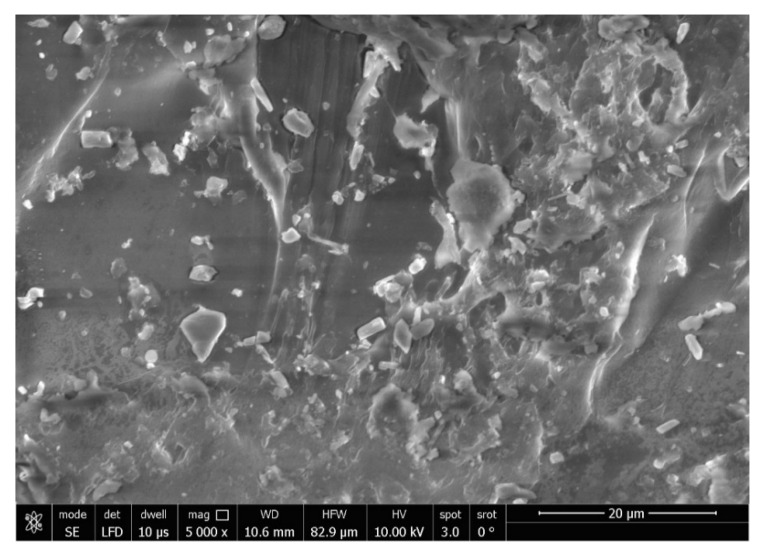
SEM micrograph of control specimen (magnification 5000×).

**Figure 8:**
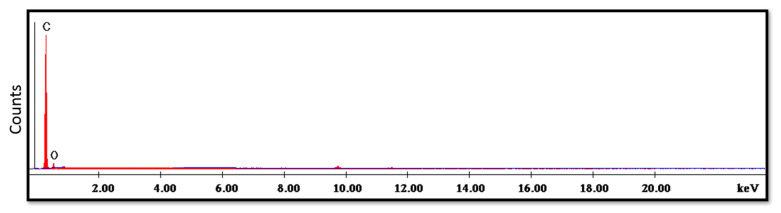
EDX spectrum of control specimen.

The SEM analysis of the control specimens are represented in Figure 6 and Figure 7. The control specimens showed surface irregularities. The elemental analysis revealed that the composition of the control specimens was mainly C and O (wt.%: 62% and 38%, respectively, or as at.%: 68% and 32%, respectively) (see Figure 8).

**Figure 9:**
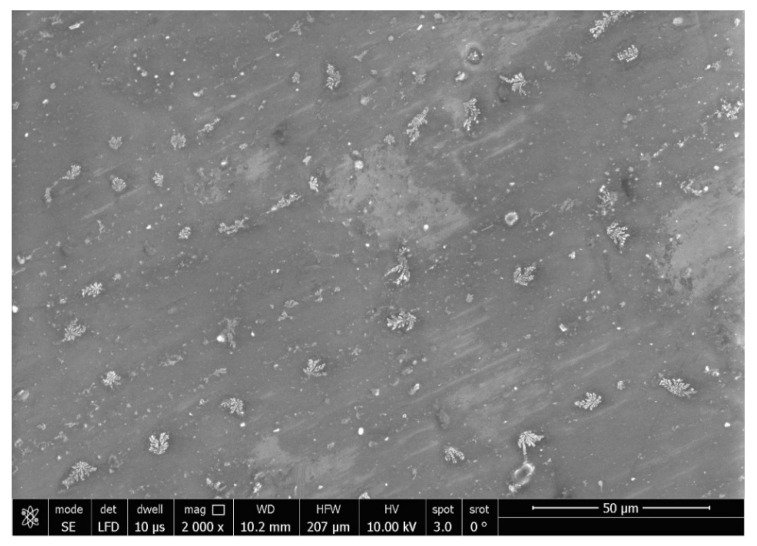
SEM micrograph of treated specimen (magnification 2000×).

**Figure 10:**
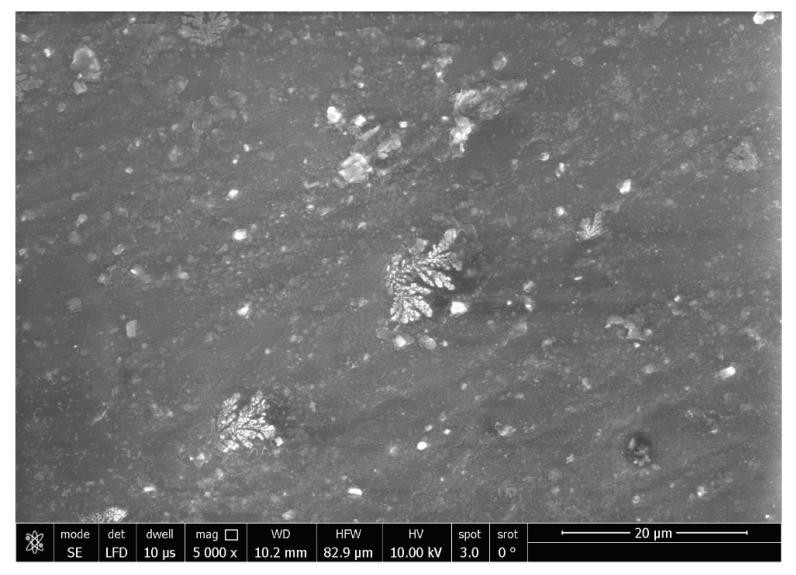
SEM micrograph of treated specimen (magnification 5000×).

**Figure 11:**
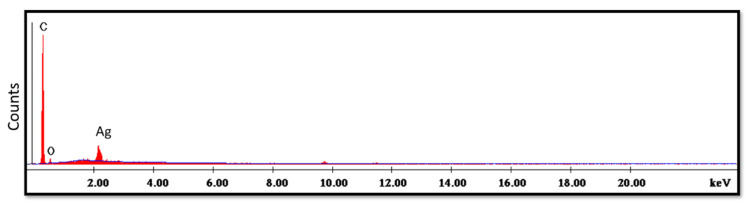
EDX spectrum of treated specimen.

The SEM micrograph of the treated specimens (Figure 9) showed an even distribution of the Ag-doped carbon nanotubes within the polymer. The higher magnification (Figure 10) displayed a leaf-like appearance of this added filler with a composition (detected using EDX) mainly consisting of C, O and Ag (Figure 11) (wt.%: 61.0%, 38.0% and 1.0%, respectively, or as at.%: 67.8%, 31.8% and 0.4%, respectively).

The impact strength, surface microhardness and agar diffusion test results against *Candida albicans* can be seen in Table 1, for the heat-cured PMMA (control group) and the treatment group with Ag-doped CNT.

### 3.2. Impact Strength

The impact strength of the treated heat-cured PMMA with the Ag-doped CNT (2.2 kJ/mm^2^) was significantly higher than the control heat-cured PMMA (1.6 kJ/mm^2^).

### 3.3. Micro-Hardness

The Vickers microhardness value of the treatment group (52.7 VHN) was significantly higher than that of the control group (19.4 VHN).

### 3.4. Anti-Candida Effect

**Figure 12:**
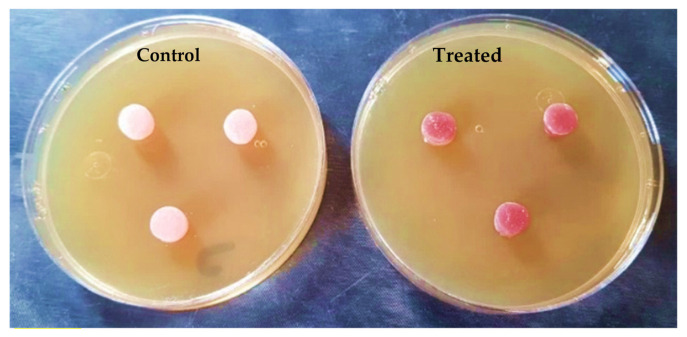
Specimens during the agar diffusion test immediately after insertion.

**Figure 13:**
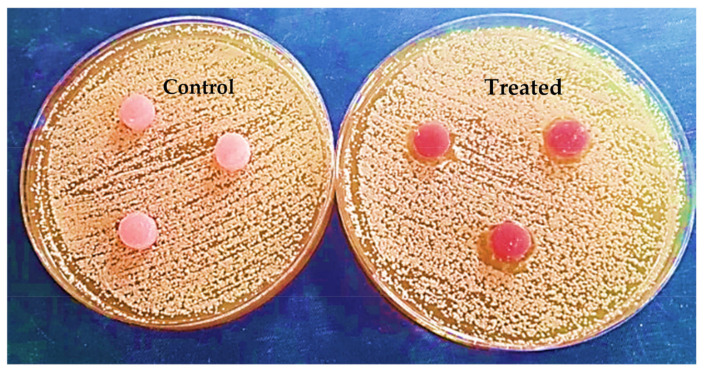
Specimens in agar diffusion test after 24 h.

The outputs of the agar diffusion test for the control and treatment groups are shown in Figure 12 and Figure 13, immediately after and 24 h after inserting the discs in plates containing *Candida albicans*.

After 24 h, there was a significant difference between the anticandidal activity of both groups. The control group did not display any inhibition zone around the discs. Meanwhile, the treatment group exhibited an inhibition zone around the peripheries of the treated discs (2.7 mm).

## 4. Discussion

Despite the desirable properties of PMMA as a conventional denture base material, fractures are very commonly encountered under impact. Therefore, a strong demand exists to enhance the mechanical properties of traditional PMMA-based, heat-cured denture bases [45]. Increasing the surface microhardness is important to prevent bacteria and fungi from adhering to the denture surface, which can lead to candidiasis and inflammation [46]. 

Moreover, the low surface microhardness of heat-cured acrylic denture bases makes them more susceptible to scratching and the formation of microcracks, weakening the denture base and promoting the growth of unwanted microorganisms [47]. Surface hardness also indicates the likelihood of plaque formation and *C. albicans* adhesion, which can cause dental inflammation. Therefore, developing heat-cured denture base materials with improved impact strength, surface microhardness, and anticandidal effects could lead to innovative materials [48].

Including carbon nanotubes (CNTs) in dental materials is expected to enhance their use in dentistry and enable new functional applications because of their improved mechanical properties [49]. Combining these advancements with the addition of Ag nanoparticles is anticipated to create novel materials with enhanced mechanical and surface properties, as well as antibacterial benefits [38,39]. This doping technique is of great interest, as it can create a hybrid nanostructure that exhibits properties from both parent components [50].

In the current study, the SEM micrographs of the produced Ag-doped carbon nanotubes revealed an agglomerated structure with a flake-like appearance. The higher magnification (20,000×) showed that the nanotubes were present in a cluster form rather than as separate nanoparticles. This was supported by a previous study that reported several large agglomerates of carbon nanotubes and stated that it was impossible to achieve a uniform dispersion of carbon nanotubes, regardless of the mixing methods or parameters, the inclusion of surfactants, or the use of various solvents. The EDX analysis revealed that the composition of the powder was mainly Ag and C, representing the doping material and nanotube material, respectively [51].

The SEM analysis of the control specimens showed surface irregularities which may denote insufficient polishing. On the other hand, the elemental analysis revealed that the composition of the control specimens was mainly C and O, referring to the chemical composition of PMMA, as the monomer is methyl methacrylate with the formula CH_2_=C(CH_3_) COOCH_3_. The EDX’s limitations in detecting hydrogen were due to the single electron present in the hydrogen atom K-shell, which is a valence electron that participates in chemical bonding. As a result, it lacks the core electron required for EDX analysis.

The SEM micrographs of the treated specimens displayed a leaf-like appearance of carbon nanotubes, which may be due to their tendency to agglomerate when impregnated in a polymer matrix which was subjected to the heat from the curing cycle [51]. It was reported previously that raising the temperature led to a uniform distribution of carbon nanotubes and a change in their morphology which changed from a flake to a leaf-like appearance [52].The EDX spectra showed mainly C as a constituent element in both the polymeric matrix and carbon nanotubes, O from the polymeric matrix—and Ag as a doping element in nanotubes.

Since the results of this investigation indicate that the addition of 0.05 wt.% Ag-doped CNT nanoparticles to PMMA resulted in higher impact strength, surface microhardness, and enhanced anti-candida activity compared with the control group, the null hypothesis must be rejected.

The improved impact strength observed in the modified PMMA can be attributed to the presence of Ag-doped CNT nanoparticle fillers, which exhibited good adhesion to the resinous matrix. This strong interfacial bonding between the matrix and fillers prevents crack propagation and allows efficient stress transfer from the weaker polymer matrix to the robust fillers [53].

The increase in surface microhardness can be explained by the presence of the homogenous distribution of hard CNT nanoparticles within the acrylic denture base materials.

The addition of Ag-doped CNT nanoparticle fillers enhanced the anti-candida activity of the specimens, as the Ag nanoparticles exhibited a bactericidal activity against *C. albicans* by disrupting their outer cell membrane and preventing microbial colonization of prostheses [54,55,56,57]. This small inhibition zone (just a few mm) of the poly (methyl methacrylate) with Ag-doped carbon nanotubes may suggest a slow release of the silver ions from the polymer composite. Thus, it is recommended to study its sustained release. Although the anti-candidal effect of the treated denture base material with the silver-doped carbon nanotube was 2.7 mm, this could be enough of a distance to affect the alveolar mucosa intra-orally due to the intimate contact between the denture base and mucosa. 

However, it is important to note that manual blending of the powders used in this research may have limitations. Furthermore, additional studies are recommended to assess any potential color changes that may be easily detected visually. Additionally, cytotoxicity tests should be conducted to evaluate any potential hazards before clinical application trials. Moreover, evaluating the flexural strength of the denture base is recommended to provide further insights into the possible reinforcement capabilities of innovative fillers during clinical service.

## 5. Conclusions

Silver-doped carbon nanotubes could be considered a promising filler for dental heat-cured acrylic resin to improve the resistance of the resultant denture against sudden fractures, scratching, and *C. albicans* invasion. However, further studies are recommended to characterize this filler type and assess the effect of its addition on the other properties of denture base materials. 

## Figures and Tables

**Table 1:** Results of various tests for the control and treatment groups.

Test	Control Group	Treatment Group	*p* Value (Sig.)
Impact strength (kJ/mm^2^)	1.6 ^a^ ± 0.2	2.2 ^b^ ± 0.1	*p* = 0.0001 *
Microhardness (VHN)	19.4 ^a^ ± 1.6	52.7 ^b^ ± 1.5	*p* = 0.0001 *
*C. albicans* inhibition zone after 24 h (mm)	0 ^a^	2.7 ^b^ ± 0.5	*p* = 0.0001 *

P: probability of the result occurring by chance. Different superscript letters (^a,b^) indicate significance * where *p* ≤ 0.05.

## Data Availability

The data presented in this study are available on request from the corresponding author.
